# A Temnospondyl Trackway from the Early Mesozoic of Western Gondwana and Its Implications for Basal Tetrapod Locomotion

**DOI:** 10.1371/journal.pone.0103255

**Published:** 2014-08-06

**Authors:** Claudia A. Marsicano, Jeffrey A. Wilson, Roger M. H. Smith

**Affiliations:** 1 Departamento de Ciencias Geológicas, Facultad de Ciencias Exactas y Naturales, Universidad de Buenos Aires, Buenos Aires, Argentina; 2 Museum of Paleontology and Department of Earth and Environmental Sciences, University of Michigan, Ann Arbor, Michigan, United States of America; 3 Department of Karoo Paleontology, Iziko South African Museum, Cape Town, South Africa; 4 Department of Geological Sciences, University of Cape Town, Cape Town, South Africa; University of Utah, United States of America

## Abstract

**Background:**

Temnospondyls are one of the earliest radiations of limbed vertebrates. Skeletal remains of more than 190 genera have been identified from late Paleozoic and early Mesozoic rocks. Paleozoic temnospondyls comprise mainly small to medium sized forms of diverse habits ranging from fully aquatic to fully terrestrial. Accordingly, their ichnological record includes tracks described from many Laurasian localities. Mesozoic temnospondyls, in contrast, include mostly medium to large aquatic or semi-aquatic forms. Exceedingly few fossil tracks or trackways have been attributed to Mesozoic temnospondyls, and as a consequence very little is known of their locomotor capabilities on land.

**Methodology/Principal Findings:**

We report a ca. 200 Ma trackway, *Episcopopus ventrosus*, from Lesotho, southern Africa that was made by a 3.5 m-long animal. This relatively long trackway records the trackmaker dragging its body along a wet substrate using only the tips of its digits, which in the manus left characteristic drag marks. Based on detailed mapping, casting, and laser scanning of the best-preserved part of the trackway, we identified synapomorphies (e.g., tetradactyl manus, pentadactyl pes) and symplesiomorphies (e.g., absence of claws) in the *Episcopopus* trackway that indicate a temnospondyl trackmaker.

**Conclusions/Significance:**

Our analysis shows that the *Episcopopus* trackmaker progressed with a sprawling posture, using a lateral-sequence walk. Its forelimbs were the major propulsive elements and there was little lateral bending of the trunk. We suggest this locomotor style, which differs dramatically from the hindlimb-driven locomotion of salamanders and other extant terrestrial tetrapods can be explained by the forwardly shifted center of mass resulting from the relatively large heads and heavily pectoral girdles of temnospondyls.

## Introduction

**Figure 1:**
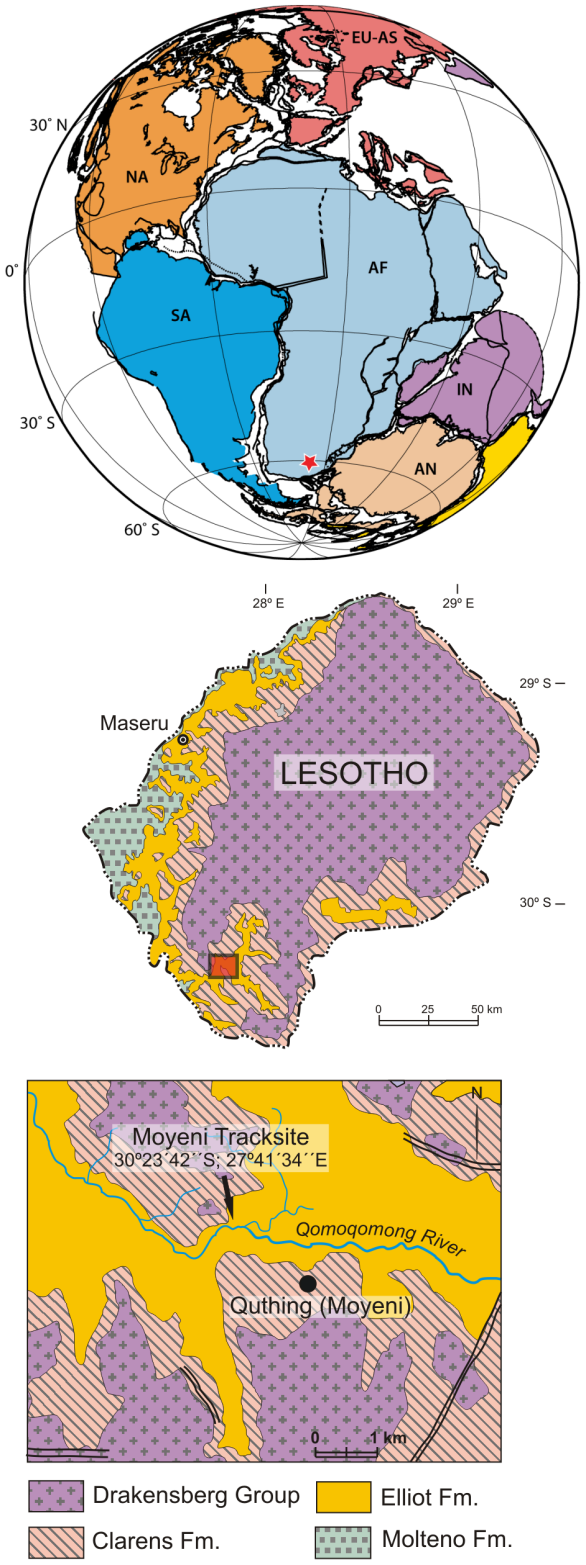
Location of Moyeni tracksite, Lesotho. Top, paleogeographic reconstruction of southern Pangea during the Late Triassic, 200 Ma (map produced at the University of Texas Institute for Geophysics, 2012; http://www.ig.utexas.edu/research/projects/plates/). Locality is indicated by a star. Bottom, Google Earth image of southern Lesotho showing location of Moyeni tracksite (building indicated by arrow) just outside the town of Quthing. **A2** and **A4** are major roadways leading to Quthing. Abbreviations: **AF**, Africa; **AN**, Antarctica; **EU-AS**, Eurasia; **IN-MA**, Indo-Madagascar; **NA**, North America; **SA**, South America.

**Figure 2:**
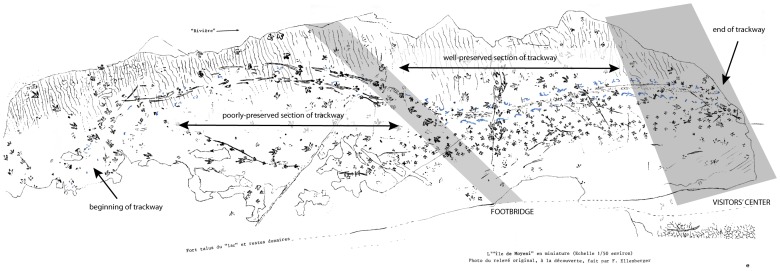
Moyeni tracksite. Track surface exposed during 1960s and 1970s, with original interpretations of tracks and surface morphology by Ellenberger ([3]; foldout e). Current position of footbridge and Visitor Center are shown schematically by translucent gray polygons. The trackway of *Episcopopus ventrosus* is highlighted in blue, and the first and last recorded steps are indicated.

The Moyeni tracksite in Lesotho, southern Africa, preserves a diverse assemblage of large- and medium-sized tetrapod tracks (Fig. 1). The site lies within the lower part of the upper Elliot Formation of the Stormberg Group, whose age has been generally accepted as Early Jurassic based on correlations of its ichnotaxa with those of North America [1] as well as on the comparative evolutionary grade of its tetrapod fauna [2]. The Moyeni tracksite was first described by Paul Ellenberger [3], who mapped the surface and identified numerous tetrapod trackways made by several trackmakers preserved in what he interpreted as an emergent sandbank formed at the mouth of a river flowing into a large lake (Fig. 2). The Moyeni tracksite constitutes one of best preserved and diverse tracksites from the Triassic-Jurassic in Gondwana.

During geological and paleontological field work at Moyeni from 2005 to 2008, we reinterpreted the depositional setting of the tracksite as a meandering fluvial channel on the lower slope of a point bar [4] and studied the locomotor capabilities of dinosaur trackmakers relative to differences in substrate inclination and slipperiness [5]. The footprints at Moyeni were imprinted into cohesive, fine-grained sand on an exposed scroll bar covered with ripples formed under rapidly waning flood flow. As water levels dropped, the shallow water flowing over the bar top washed-out the ripples and replaced them with current lineation. The flanks of the bar remained sculpted by low amplitude, small current ripples orientated tangential to slope. These surfaces were subsequently draped with a veneer of silty clay that settled out of suspension. A short period of subaerial exposure of the bar is evidenced by wrinkle marks in the claystone veneer, run-off rills, and a distinctive, pustular, surface texture imprinted by shrinkage of a microbial mat that was lowered onto the bar surface [4].

In addition to saurischian and ornithischian dinosaurs, non-dinosaur tetrapod tracks have been identified at Moyeni. *Episcopopus ventrosus* tracks were made by a large, slow-moving quadruped with a low body carriage (see [6], p. 347; pl. 6, fig. 70). The generic name is derived from the Greek word *episkopus*, which refers “bishop” according to Ellemberger ([3], p. 53) due to the resemblance between the arcuate limb drag marks and a bishop's crook. The specific name refers to the trackmaker's “heavy belly” (*venter*, Latin), which flattened the ground below it. The brevity of the initial description of *Episcopopus* and other Stormberg ichnotaxa [6] has led some to regards several of these ichnotaxa as *nomina nuda* [7]. However, given the detailed description provided later [3], we agree with Porchetti & Nicosia [8] that “following the spirit of the meaning of priority as well as the ethics of scientific propriety,” the ichnotaxa established by Ellenberger (1970) should be “considered as valid in principle but in need of amendment” (p. 221).

*Episcopopus ventrosus* is a rare and enigmatic ichnotaxon that has only been reported from the Moyeni tracksite, where only a single, long trackway was recorded. There are no *Episcopopus*-like trackways known from elsewhere in Lesotho, southern Africa, or indeed from Pangea—not even from the Newark Supergroup, which shares many ichnotaxa with Moyeni [1]. The identity of the *Episcopopus* trackmaker has remained enigmatic. Ellenberger [3], [6] entertained the possibility of crocodilian, chelonian, or even dinosaurian trackmakers, but subsequent interpretations narrowed the identity to a basal chelonian [1], [9].

In this paper, we describe the ichnomorphology of the *Episcopopus ventrosus* trackway, which we attribute to a ca. 3.5 meter-long temnospondyl. Our re-investigation of *Episcopopus* used a combination of traditional mapping and laser-scanning techniques. The high-resolution surface scans revealed for the first time details of the footprints and belly drag. We conclude that *Episcopopus* represents the first definitive temnospondyl trackway from the Mesozoic and provides useful comparisons with the locomotor patterns of extant salamanders, which are frequently used as the model for basal tetrapod locomotion.

## Methods

**Figure 3:**
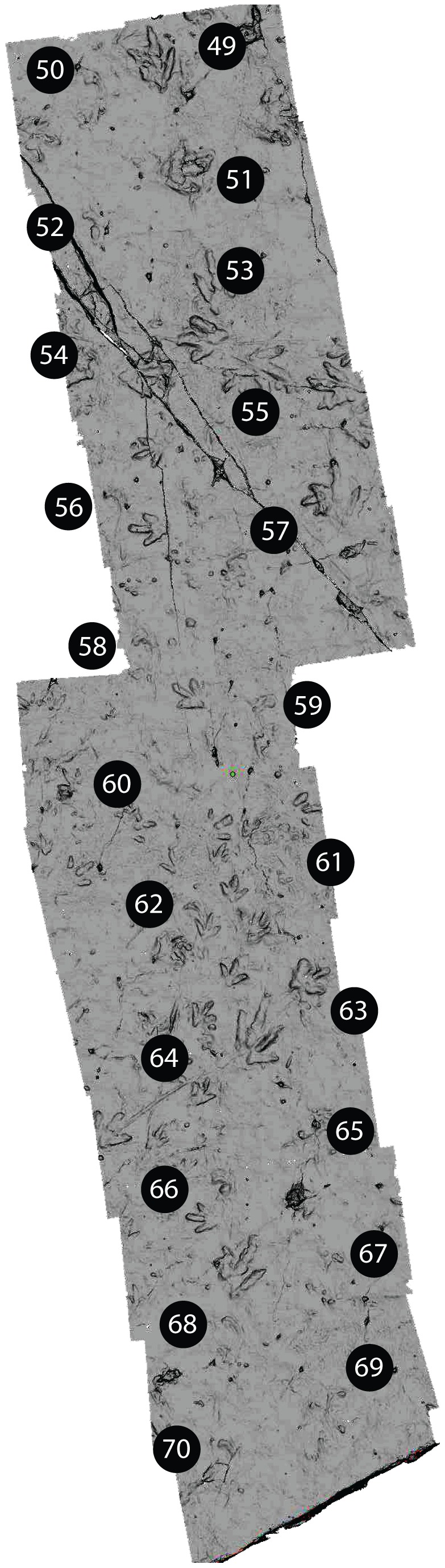
Moyeni tracksite, overview of scanned surface. This map, which includes *Episcopopus ventrosus* manus-pes pairs 49–70 (Ellenberger's [3] numbering), was constructed from a series of 50×50 cm scans.. For higher resolution images, see Figures 4–7.

The map in Figure 3 was made from three-dimensional data collected on site using a HandyScan HZ portable laser scanner at 0.63–0.88 mm resolution. Individual 50×50 cm squares were scanned, edited in Materialise Magics 14, and then stitched together in Materialise 3-Matic 4.4. Two-dimensional images were created from the modified three-dimensional data. Interpretation of track morphology was based on our in situ observations as well as images of three-dimensional data created with a controlled light source. The corresponding permits for field work in Quting area were issued by the Secretary of the Protection & Preservation Commission, Department of Culture of Lesotho.

Trackway measurements reported in Table S1 follow the standard protocols. Stride length is the distance between homologous points on successive prints of the same foot. Pace length is the distance between homologous points on the prints of the left and right manus or pes. The pace angle is formed by successive paces (i.e., R–L–R or L–R–L). Linear measurements were made to the nearest 0.5 centimeter and were measured *in situ* unless noted; angular measurements were made to the nearest 0.5 degree using the digital map of the trackway surface.

### Institutional Abbreviations

**SAM**, Iziko South African Museum, Cape Town, South Africa; **UCMP**, University of California Museum of Paleontology, Berkeley, California, U.S.A.; **UMMP**, University of Michigan Museum of Paleontology, Ann Arbor, Michigan, U.S.A.; **UMMZ**, University of Michigan Museum of Zoology, Ann Arbor, Michigan, U.S.A.

## Results

### Systematic Ichnology

Ichnogenus *Episcopopus* Ellenberger 1970

Episcopopus ventrosus

Figs. 2–7

**Figure 4:**
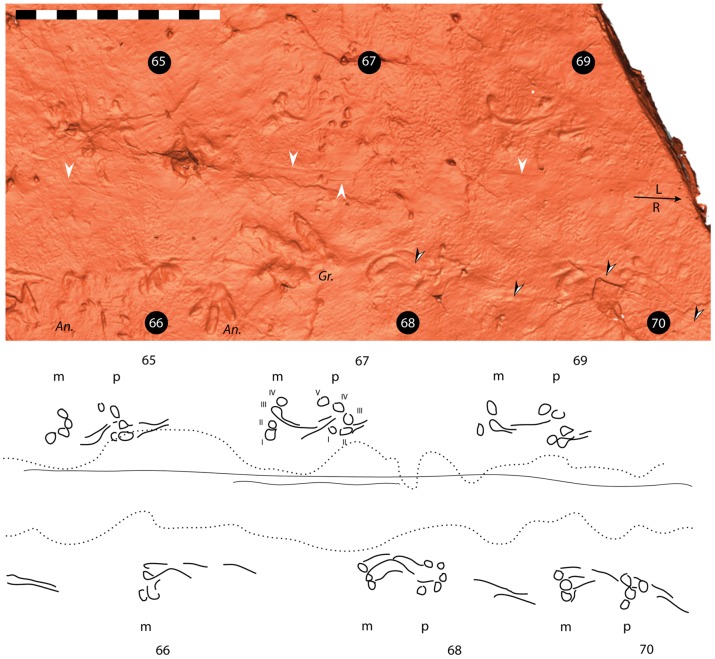
*Episcopopus ventrosus.* Scanned image (top) and interpretive line drawing (bottom) of manus-pes pairs 65–70. Odd numbers represent left manus-pes pairs; even numbers indicate right manus-pes pairs. Direction of travel and left (**L**) and right (**R**) are indicated by the arrow at right. Abbreviations: **I**–**V**, digits; ***An*** **.**, *Anamoepus*; ***Gr*** **.**, *Grallator*; **m**, manus; **p**, pes. White arrowheads indicate straight drag marks probably made by the base of the tail; black and white arrowheads indicate manual digit drag marks. Scale equals 1 m. m.

**Figure 5:**
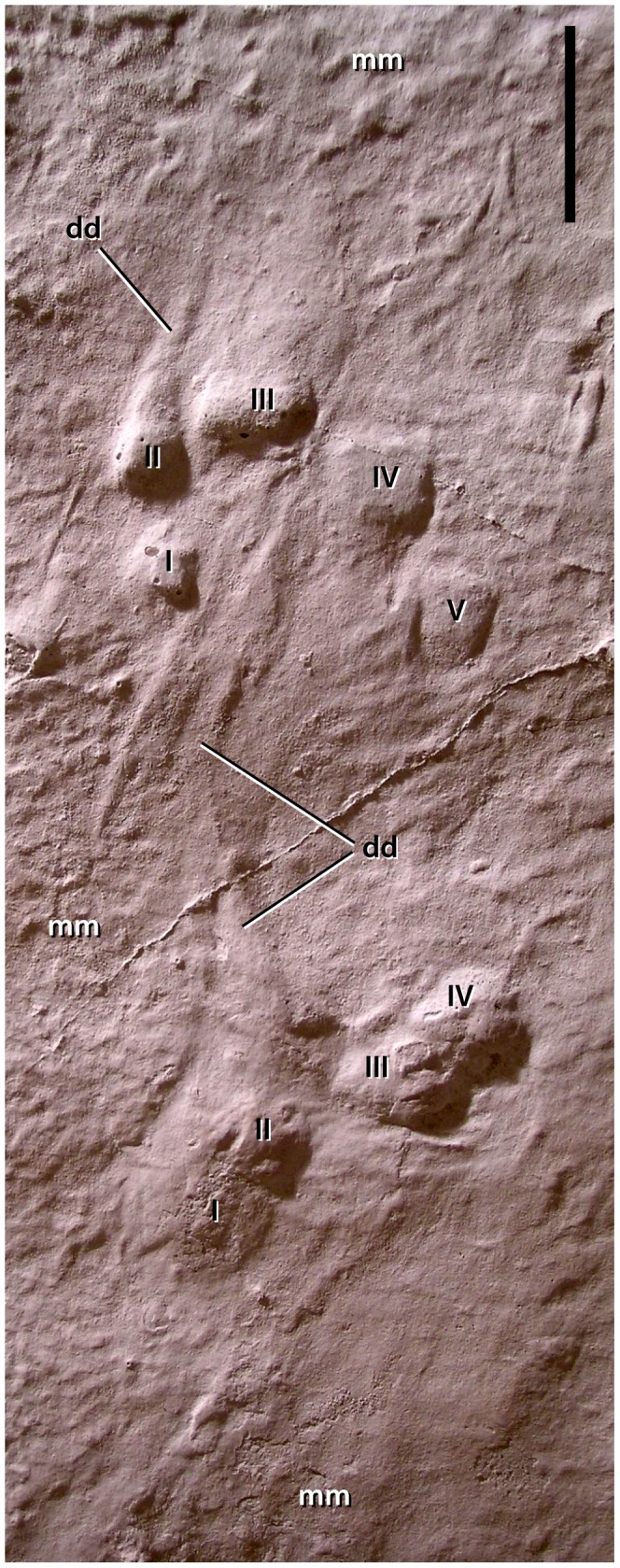
*Episcopopus ventrosus.* Right manus-pes pair 56 demonstrating the superposition of the pes print over the manus drag. Note that this is photograph of a cast (positive), with light directed from the upper left. The pentadactly pes (**I**–**V**) is positioned forward of the tetradactyl manus (**I**–**IV**) and oversteps truncates the digital drag marks (**dd**) of the manus. The microbial-matted surface (**mm**) has been smoothed in the immediate vicinty of the tracks. Scale equals 5 cm. cm.

**Figure 6:**
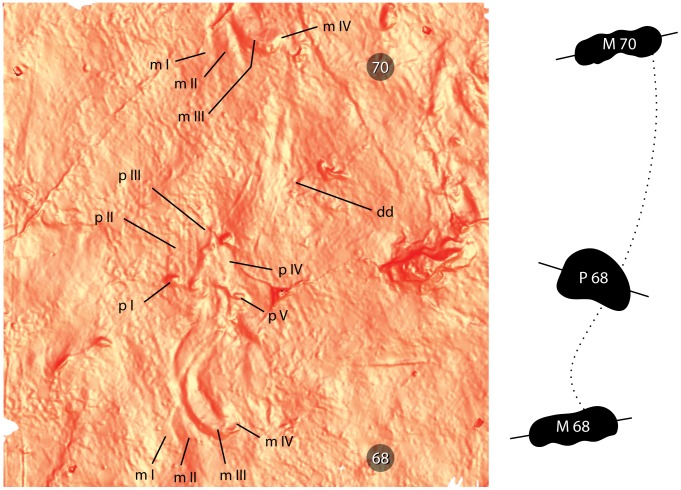
*Episcopopus ventrosus*. Sinusoidal manus drag between successive footfalls. Image at left shows right manus-pes pair 68 and and right manus 70. The rightmost edge of the belly drag, which smoothed the microbially-matted surface, can be seen at left. The interpretive drawing at right shows schematic outlines (filled in black) of the right manus and pes along with a dotted line indicating the arc of curvature of the drag. Abbreviations: **I**–**V**, digit numbers; **dd**, digit drag; **m**, manus; **p**, pes.

**Figure 7:**
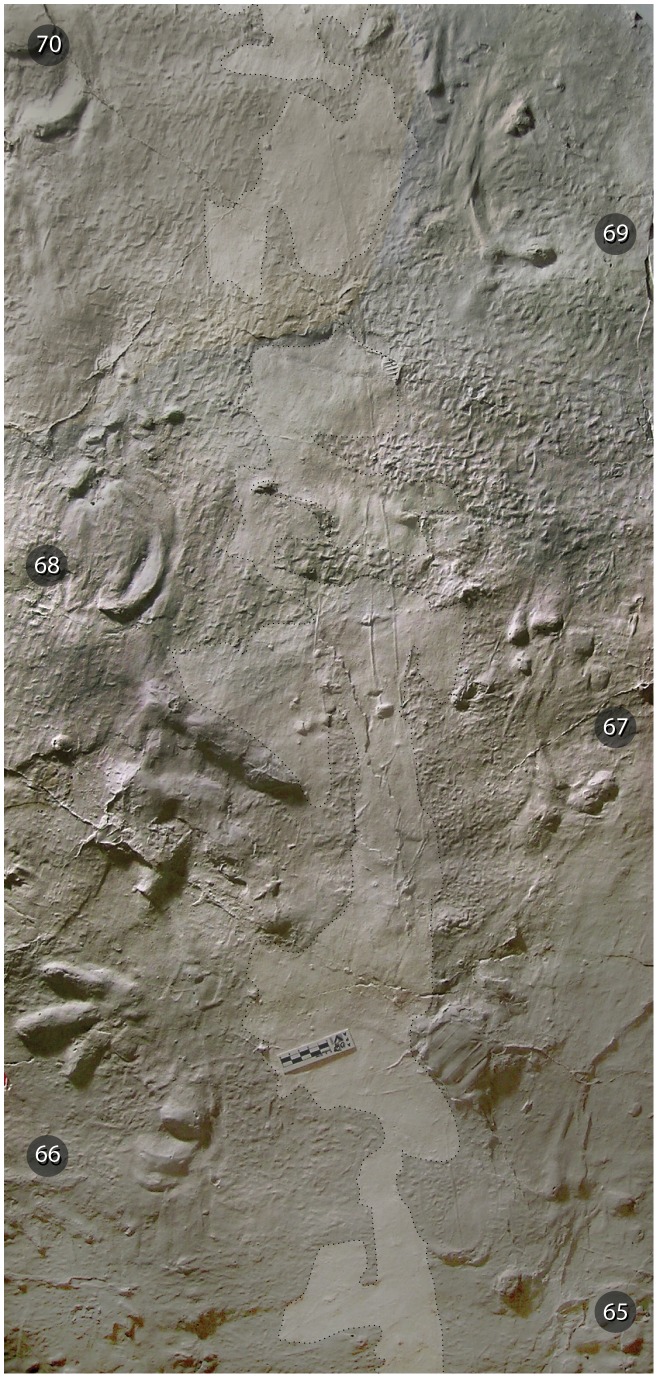
*Episcopopus ventrosus*. Belly drag between manus-pes pairs 65–70. In this photograph of a plaster cast of the trackway, made on site by the authors, the belly drag was recognized by the smoothing of the microbial-matted surface between the successive footfalls. Our interpretation, which is marked in the translucent white tone bordered by a dotted line, differs from that portrayed by Ellenberger across nearly the same interval ([3]: fig. 70, pl. I), which was continuous, broader, and slightly more regularly undulating. That is, our interpretation is that the belly drag was narrower, more discontinuous, and irregular. The scale bar is in centimeters (lower central portion of image).

#### Material

Moyeni tracksite, Quthing District, Lesotho, southern Africa. In addition to the original prints, there are plaster casts of different sections of the trackway that were made by Ellenberger (Collection Paul Ellenberger—Pistes du Stormberg, Laboratoire de Paléontologie, Institut de Sciences de l'Evolution, Université Montpellier II; [10]) and by the authors (SAM-PT-K10949). The digital archive of the northern portion of the trackway is stored at the University of Michigan Museum of Paleontology.

#### Diagnosis

ca. 3.5 m-long quadrupedal trackmaker with four manual digits and five pedal digits, all of which lack claws. The digital impressions of the manus and pes are convex anteriorly; together the manual digit impressions form a chevron shape, and together the pedal digit impressions form a rounded arch. The pes oversteps the manus of the previous stride (i.e., secondary overstepping), implying the trackmaker had a relatively long body with short limbs. The body drag is broad and lacks evidence of undulation.

Other distinctive features of *Episcopopus* may be related to behavior and substrate in this exemplar. These include the lack of registration on the surface by the proximal phalanges or metapodials, as well as the drag marks associated with manus and pes, which differ from one another. The drag marks associated with the manus are sigmoid, elongate, and distally converging, whereas those associated with the pes are short, straight, and non-converging. These latter features are not diagnostic of the ichnotaxon, but provide information about its locomotion, as discussed below.

#### Comments

Ellenberger [3], [6] made interpretations of the *Episcopopus* tracks that differ substantially from to the one we present below. Some of the more salient differences include the identification of the manus and pes, the number of digits, the presence of claws, and the interpretation of the body drag (see “Description” below).

### Description

Ellenberger [3] described 82 successive manus-pes pairs on the *Episcopopus* trackway, which he numbered sequentially (Fig. 2). According to him [3], the trackway starts subaqueously at the southern extreme of the tracksite, on the lower ripple-marked portion of the bar, and climbs tangentially up the western margin of the bar until it emerges from the water just south of the position of the footbridge that now crosses the site. We were not able to identify any definitive *Episcopopus* tracks south of the bridge as the surface has been eroded since it was studied by Ellenberger in the 1960s. At that time, only the deepest parts of the tracks were visible [3]. The *Episcopopus* trackway continues northwards from the current position of the bridge across the emergent microbial-matted bar top. This microbial mat has a uniform pustular texture [4], and surface scan images show that this part of the surface was very gently undulated by low-amplitude ripples oriented perpendicular to the trackway axis. The northernmost 3 m of the trackway, which records the last steps made by the *Episcopopus* trackmaker on this surface, is now hidden beneath the Visitors' Center building (see [3]). Thus, due to natural weathering and the new construction, our sample of the *Episcopopus* trackway is limited to 22 manus-pes pairs exposed between the bridge and Visitors' Center, which correspond to manus-pes pairs 49–70 in Ellenberger's [3] sequence (Fig. 2). Although this is a smaller sample than that studied by Ellenberger [3], the tracks that are available to us are still well preserved and offer insights into the identity and mode of locomotion of the *Episcopopus* trackmaker (Fig. 4).

The *Episcopopus* trackway records a quadruped animal with a sprawling gait, moving steadily northward (i.e., in the direction of current) with a regular stride length (ca. 110 cm) and pace angulation (ca. 72°) for both manus and pes (see Table S1). The manus and pes prints are well separated from the midline and the low-slung ventral surface of the trackmaker's body left drag marks between its footprints.

The tracks were imprinted on a microbial mat, which bears signs of subaerial exposure in the shrinkage puckering [11-13]. Dragging of the belly and digits over the microbial mat has smoothed its puckered surface (Fig. 4). We conclude that there was no standing water on the bar top when the trackmaker moved across the matted surface; thus the *Episcopopus* trackmaker was engaging in terrestrial locomotion, not aquatic locomotion or bottom-walking.

The direction of travel is deduced from the shape of the manus and pes digit impressions, both of which form an arc with the convexity facing the same direction. We assume here that the convex side of the manus and pes impressions indicates the forward direction of travel, because we know of no tetrapod has a manus or pes in which lateralmost and medialmost digits are longer than central digits, or that makes anteriorly concave manus and pes impressions. We distinguished between manus and pes impressions by their cross-cutting relationships. In a forward-moving trackmaker, the pes can superpose the manus impression, but not vice versa. Detailed observations of the trackway surface, laser scans, and plaster casts allows us to identify the pes as the more tightly-arched structure that consistently oversteps the manus and crosscuts the manus drag impressions (Fig. 5). Our identification of the manus and pes in the trackway is exactly opposed to Ellenberger's [3] interpretation, in which the manus print is positioned in front of the pes print (see “Comments” above).

**Figure 8:**
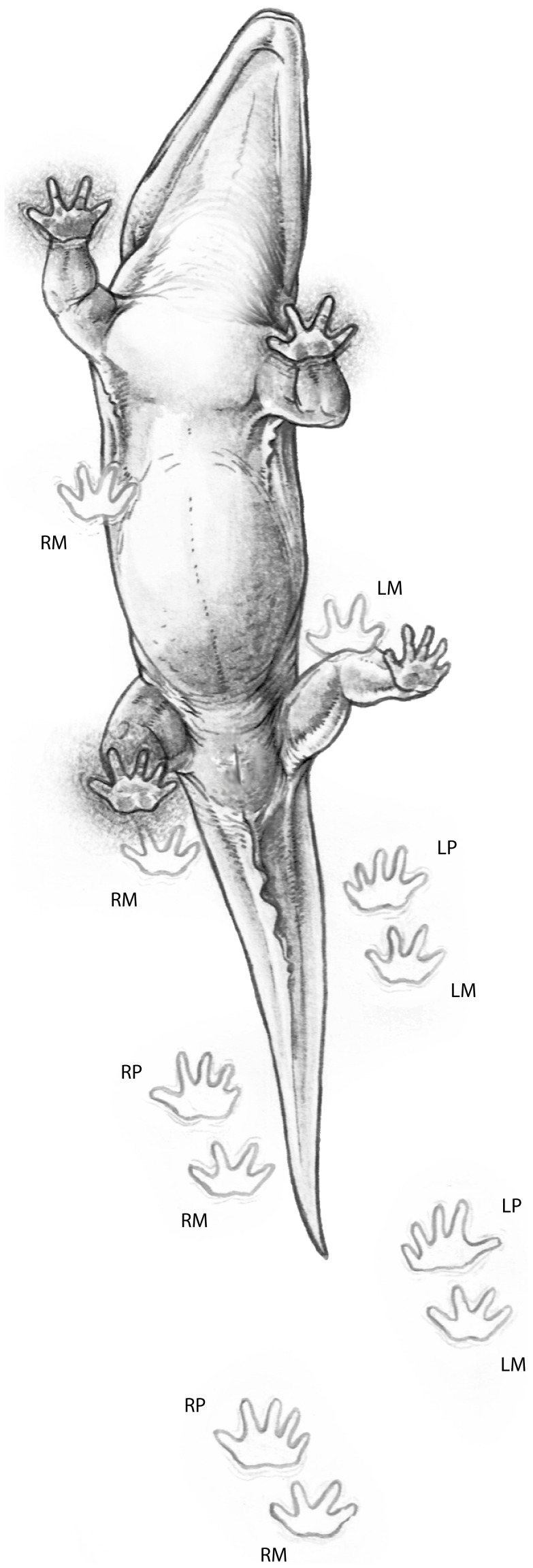
Secondary overstep in a ‘long-coupled’ tetrapod trackmaker. The illustration shows the underside of a stereospondyl as it progresses along a hypothetical trackway surface. The lower portion of the illustration shows the sequence of impressions made by the right and left manus (**RM**, **LM**) and pes (**RP**, **LP**) leading up to the moment just before the left pes contacts the substrate. Note that in this ‘long-coupled’ form the pes oversteps the manus of the previous stride. We infer that the *Episcopopus* trackmaker made tracks in this fashion.

Relative measurements of trackway width, size of the prints, and stride length were used to calculate the body size of the *Episcopopus* trackmaker (Table S1). The ca. 130 cm glenoacetabular distance and relative shortness of the limbs suggests that the pes-manus overstep is probably second phase; that is, the pes oversteps the manus of the previous stride (Fig. 8). This has been referred to as “long coupling” [14]. In this case, the fairly regular print impressions and constant trackway width (ca. 65 cm) suggest that the secondary overstep is due to the relatively short limbs in the trackmaker rather than a very long-bodied animal or a running “short coupled” gait, as noted by Peabody [14] in his study on salamander locomotion.

The manus prints are preserved as shallow impressions of the tips of the digits (I to IV) and their associated drag marks. No digit impression or drag marks are preserved lateral to these, so we conclude that the trackmaker had a four-digit manus. Occasionally, only the drag marks of the digits are present (e.g., manus print number 68). Not a single palm impression was observed in any of the 22 manus prints studied, nor were they noted by Ellenberger [3] in the rest of the trackway. It follows that either the palm did not contact the substrate, or it made contact but did not bear enough weight to imprint the surface, which preserves digit drag marks. The digits are clawless, with gently rounded tips. The combined impressions of the digit tips form an inwardly oriented /\-shape that is ca. 25 cm wide by ca. 15 cm long (Figs. 4–6). The apex of the /\ is formed by the digit III print. The digit IV print is located just lateral to the apex, and digits II and I are positioned progressively posterior and medial to it. The outer digits (i.e., III, IV) made deeper, more distinctive impressions than did the inner digits (i.e., I, II). Consequently, in the less well preserved, shallower prints, it is the outer digits, especially digit IV, that are most readily identified (e.g., manus print 69). The impression of digit II could be consistently identified when the light source and contrast of the scanned images were manipulated, but the impression of digit I could only be identified in four instances. All digit impressions are deepest anterolaterally, perhaps as a result of pressure exerted during the “stance phase” of the step cycle [14], [15].

All manus prints have distinctive sinusoidal (i.e., “crook-shaped”), forwardly-directed drags for which the genus name of the track was derived (see above; Figs. 2–6). These drag marks were created during the “swing phase” of the step cycle [14], [15]. They originate from the anterior ends of digits II, III, and IV and converge just in front of the manus print. This convergence of digit impressions implies that the digits faced laterally when planted on the substrate, but superposed one another as the manus was lifted from the substrate and moved inwards at the beginning of its swing. Later, as the forelimb moved forward, the trailing digits impressed a pronounced concave arc that swung outwards before ending with a slight inward movement towards the midline. In some of the best preserved tracks, the end of these trailing digit impressions lead into to the next manus (Fig. 6). Similar S-shaped drag marks are registered by the pes of the living salamander *Taricha* sp., especially when walking on “soft” substrates or when the animal is fatigued [14].

*Episcopopus* pes prints record only the impressions of the tips of five digits; no trace of the sole could be detected in any of the scans. These digit impressions are rounded to somewhat laterally elongated, and like the manual digits, they lack claws. Typically, all five digits are recognizable, and the impressions of digits II–V are arranged in an arc that is ca. 20 cm wide by 20 cm long. The arc is orientated slightly inwards, with the digit I imprinted just posterior to digit II. The interior three digits (i.e., II–IV) make deeper, more distinct impressions, whereas digits I and V make more subtle impressions. Digit I in particular is commonly only visible after the light source and contrast are adjusted. Similar to the manus digits, the pedal digit prints deepen anterolaterally and they also have trailing digit impressions. However, these are shorter, weakly impressed in the sediment, and only associated with digits II and III (Fig. 5). The pedal digit drags are anteriorly directed and run nearly parallel to one another without converging distally. There is no discernible curvature to the pedal digit drag marks, which could be attributed to either their relative shortness or more likely to the absence of significant lateral motion of the pes as it was lifted from the substrate (Figs. 4–6).

The *Episcopopus* trackway is also characterized by the presence of a median body drag mark (ca. 40 cm wide), which is particularly evident on the microbial-matted bar top surface (Figs. 4, 7). The belly drag was described by Ellenberger [3], who considered it a diagnostic character of the ichnospecies and attributed its apparent alternating lobes to “balancing” of the animal body during progression. The casts and laser scans demonstrated that the alternating lobes of the body drag were the result of the trackmaker's ventral surface contacting the crests of low-angled ripples (Fig. 7). The ripples cover the paleosurface (see above) and are oriented perpendicular to the axis of elongation of the bar form and tangential to the direction of locomotion of the *Episcopopus* trackmaker. Thus, variations in the width and depth of the belly drag traces are due to undulations in the surface topography rather than any vertical or lateral movement of the body.

Close to the middle of the broad, shallowly impressed body drag, there is a long, narrow groove that is flanked by a shorter groove along part of its length. Ellenberger [3] interpreted these as tail impressions, perhaps due to their resemblance to the broad, shallow furrow with lateral ridges made by the tail of ambystomatid salamanders [14]. Another possible explanation is that the elongate groove was made by a single ridge of the tail, but the tip of the tail occasionally contacted the substrate to create a parallel groove. The linear form of these ridges suggests that the proximal portion of tail, which made the drags, was not swinging from side to side. In his study on living salamander trackways, Peabody [14] observed that even when undulatory movement of the body is not evident in the trackways, its presence can be detected by lateral undulation of the tail. Nevertheless, in *Episcopopus*, both the body and tail drags lack lateral undulation, indicating that the belly and tail were moving forwards in a straight line when in contact with the sediment. The only evidence of lateral undulation in the locomotion comes from the marked S-shaped manus drags. Although there are short drag marks associated with the pedal digits, they do not indicate any sideways movement of the pes during the step cycle. Thus it appears that only the forelimbs were strongly swung inwards and then outwards during progression.

### Trackmaker Identification

In the original description of *Episcopopus ventrosus*, Ellenberger [6] identified the trackmaker as a primitive crocodilian or possibly a turtle. Later, in his monograph on the footprints and trackways from Lesotho, Ellenberger [3] provided a full description of *Episcopopus* and attributed it to a basal crocodilian, based on the resemblance to body fossil taxa described from the same stratigraphic interval (“Upper Stormberg”). Although Ellenberger [3] could not rule out the possibility that the *Episcopopus* trackway was made by an ornithischian dinosaur, he rejected his previous hypothesis of a chelonian trackmaker. Later, Olsen and Galton [1] re-evaluated Ellenberger's ichnotaxonomy of Upper Stormberg footprints, synonomizing 19 of his ichnotaxa with forms from the Newark Supergroup, identifying 26 as indeterminate, and recognizing only one, *Episcopopus ventrosus*, as “distinct but possibly indeterminate” (p. 94). Olsen and Galton [1] recognized a superficial similarity to chelonian tracks, which was supported by the presence of proganochelyid body fossils in the same strata [16]. Knoll [9] likewise suggested an austrochelyid trackmaker for *Episcopopus* tracks.

**Figure 9:**
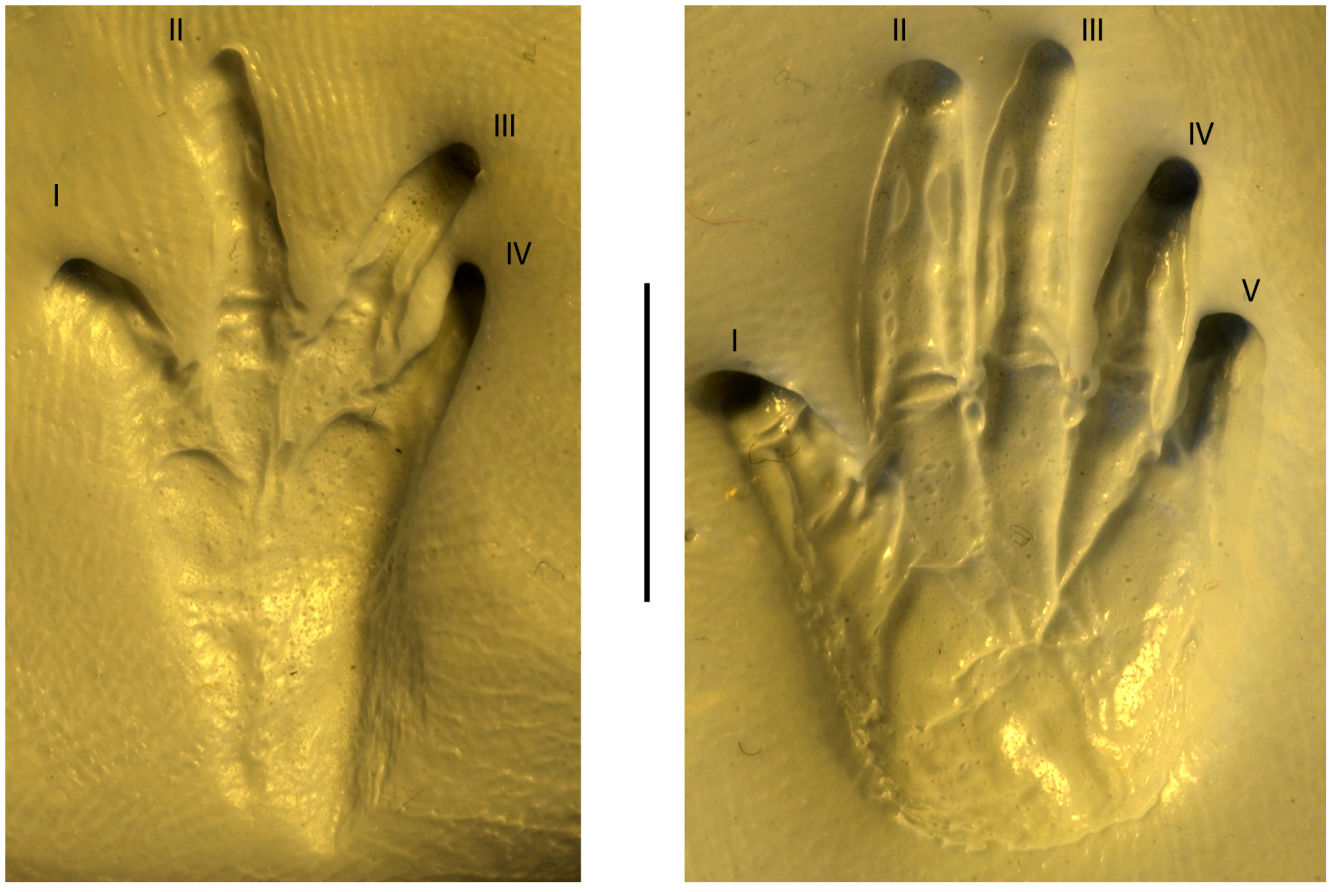
Salamander manus and pes surface morphology. Photographs show the impressions left by the undersides of the right manus and pes of the salamander *Dicamptodon ensatus* (UMMZ 135631). Roman numerals indicate digit number. Scale equals 1 cm. cm.

Our approach to trackmaker identification hinges on recognizing features preserved in the tracks and trackway that indicate body fossil morphology (i.e., manus, pes, abdomen, tail) and using those proxies to identify the trackmaker clade [17]. Synapomorphies and symplesiomorphies preserved in *Episcopopus* suggest that it was produced by a quadrupedal basal tetrapod, most probably a temnospondyl. Symplesiomorphies include wide-gauge, sprawling gait, and belly drag, the latter of which results from the combination of a relatively long, stout body and short limbs. In addition, the sinuous impressions made by the trailing manual digits support the hypothesis that the trackmaker did not walk with an erect posture. This type of progression is primitive for tetrapods but is also present in some groups that have secondarily adapted to a semi-aquatic mode-of-life, such as modern crocodilians [18]. It is absent in therapsids and dinosaurs, thus excluding them as potential trackmakers. The absence of claws in both manus and pes is another symplesiomorphy present in amphibians (Fig. 9) that indicates a basal tetrapod trackmaker and excludes amniote trackmakers such as crocodilians and turtles. The presence of a four-digit manus and five-digit pes is a synapomorphy of Amphibia (i.e., Temnospondyli + Lissamphibia) and is also present in some Paleozoic lepospondyls (microsaurs, nectridians) and the Carboniferous colosteid stem-tetrapods [19], [20]. Although the absence of toe impressions in footprints can be a taphonomic anomaly, we are confident that a fifth manual digit was not present in the *Episcopopus* trackmaker because high resolution laser scanning was unable to detect even the most subtle impression at either the medial or lateral edge of the manus. We discount nectridean and microsaur lepospondyls as potential trackmakers because they are much smaller than the *Episcopopus* trackmaker. The largest microsaurs are ca. 10x smaller, and the largest nectrideans are ca. 5x smaller; they are also ca. 50 Ma older than the Triassic-Jurassic age of the Moyeni tracksite, which would imply a lengthy ghost lineage.

In light of this combination of synapomorphies, symplesiomorphies, and by process of elimination, the most likely candidate trackmaker is a relatively large temnospondyl amphibian. During the Mesozoic, temnospondyls were represented by the stereospondyls, a highly diverse clade of semi-aquatic to aquatic amphibians [21]. Their aquatic mode of life is characterized by the presence of relatively small and poorly ossified limbs. It is probable that aquatic stereospondyls were incapable of elevating their ventral surface from the ground during locomotion. In addition, the well-defined manus drag-marks are consistent with an animal that had a forwardly-located center of mass and bore more weight on its anterior limbs, due to a relatively large skull and heavy pectoral girdle.

**Figure 10:**
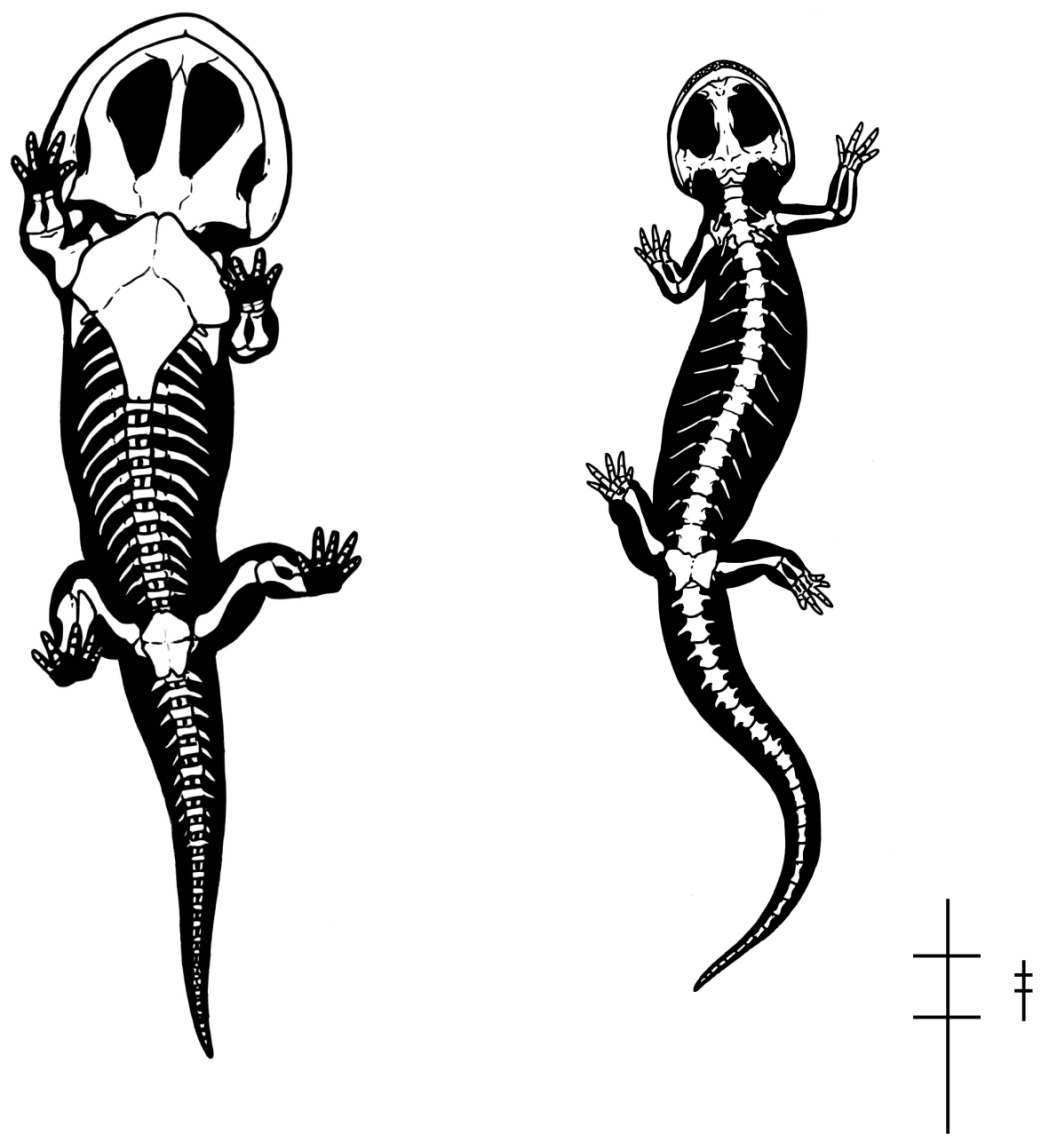
Comparison of the body shape in a *Siderops*-like temnospondyl (left), which is inferred to be the *Episcopopus* trackmaker, and a salamander (right). Skeletal silhouettes are shown in ventral view. We calculated the glenoacetabular distance for the temnospondyl to be ca. 130 cm, and its body form is based on comparisons with complete skeletons of Mesozoic stereospondyls, such as the Australian chigutisaurid *Siderops* [22] and the European capitosauroid *Mastodonsaurus* [23]. The salamander body is based on *Dicamptodon ensatus* (UMMZ 135631) and skeletal information from http://digimorph.org/specimens/Ambystoma_tigrinum/whole/. Important differences between these two include relative size and ossification of the skull and pectoral girdle, robustness of dorsal ribs, and degree of ossification of carpal, tarsal, and metapodial elements. Silhouette reconstructions are not to scale; see schematics at the bottom right size proportions.

We estimate the total body length of the *Episcopopus* trackmaker to be approximately 350 cm (Fig. 10), based on the calculated glenoacetabular distance (ca. 130 cm) and comparisons with complete skeletons of Mesozoic stereospondyls, such as the Australian chigutisaurid *Siderops* [22] and the European capitosauroid *Mastodonsaurus* [23]. Cranial fragments of a large, *Siderops*-like chigutisaurid were found in strata from South Africa [24] that are coeval with the Moyeni tracksite.

### Mesozoic Temnospondyl Track Record

The rarity of trackways attributed to temnospondyls in the Mesozoic, particularly during the Triassic, contrasts with the relative abundance of their body fossils, which comprise ca. 160 genera described from Mesozoic levels [23]. Until now there have been no definitive Mesozoic temnospondyl ichnofossils, despite descriptions of several putative temnospondyl tracks, discussed below.

#### Capitosauroides bernburgensis

**Figure 11:**
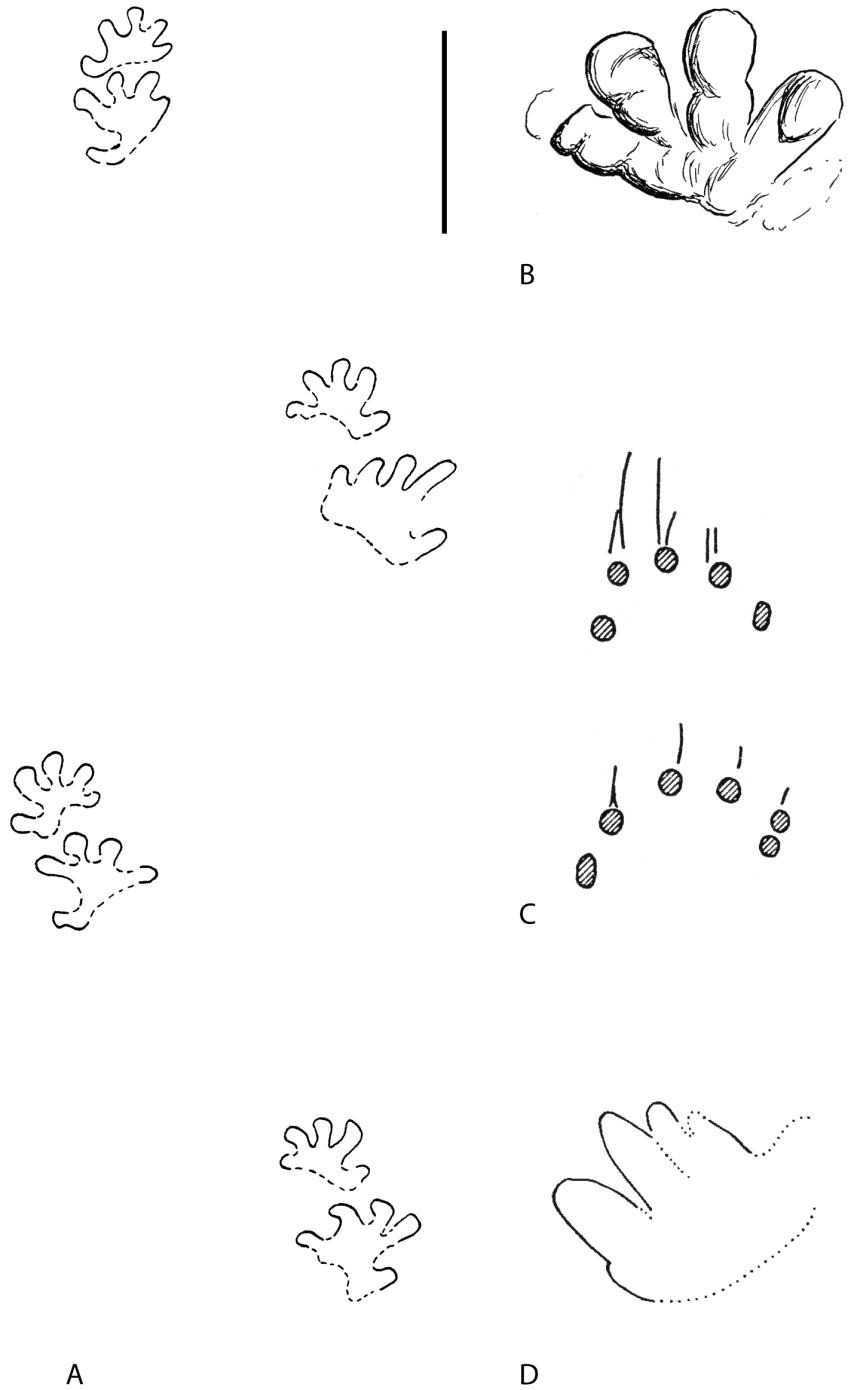
Putative Mesozoic temnospondyl tracks. **A**, aubold (1971); **B**, Pohlig (1893); **C**, Peabody (1948); **D**, Fuglewicz et al. (1990). Scale bar equals 10 cm for **A** and **C**; 5 cm for **D**. The original image in **B** was published without a scale.

The first Mesozoic temnospondyl footprints were reported from Lower Triassic exposures in Germany [25], [26]. The isolated footprint figured by Spitz [26] (Fig. 11B) was discovered on a geological excursion during a conference at Tübingen attended by F. von Huene, E. Hennig, O. Abel, and F. Nopcsa. It was collected from an Upper Buntsandstein outcropping near Kappel, Baden-Württemberg (southern Germany). Apart from the field sketch (drawn by Huene), no description was provided, and the track was not given a name. According to Spitz [26], Hennig attributed the print to a temnospondyl trackmaker based on the presence of skeletal remains of the temnospondyl *Mastodonsaurus cappelensis* in the same unit.

Haubold [27], [28] later described two trackways, one with 5 manus-pes pairs and the other with 3 manus-pes pairs, from slightly lower strata (Middle Buntsandstein) exposed at Bernburg near the Saale River in central Germany (Fig. 11A). Haubold [27] created the new ichnogenus and ichnospecies *Capitosauroides bernburgensis* to encompass the new trackways, as well as the isolated tracks reported earlier [25], [26] because they were similar in form and provenance. All the German *Capitosauroides* tracks appear to lack claws. The trackways, in which the manus and pes can be unambiguously identified, were reported to preserve a pentadactyl manus. However, the presence of the lateral-most manual digit (i.e., digit V) is difficult to confirm from available photographs (see [27], pls. 1–2). Although most temnospondyls have a tetradactyl manus, Haubold [27] nonetheless attributed the tracks to that group, citing examples of temnospondyls that were at that time restored with five manual digits.

Fuglewicz and colleagues [29] described a cf. *Capitosauroides* trackway from Middle Buntsandstein levels at Wióry, in the Holy Cross Mountains of Poland (Fig. 11D). The material includes a trackway consisting of four left manus-pes pairs plus one isolated manus and four right successive pes impressions that lack the posterior margin due to poor preservation [29]. On the left side, the manus prints are slightly smaller and positioned somewhat laterally to the corresponding pes print. The tips of the digits are clearly outlined in each print, and no metapodial impressions were observed [29]. The Polish specimen was reported to be similar to the German *Capitosauroides* tracks, but differs from them in its larger trackway width and overstepping of the manus by the pes. We could not corroborate the number of manual or pedal digits reported by Fuglewicz et al. [29] from the photographs they provided; none of the impressions appear to be clearly preserved.

The European tracks attributed to the ichnotaxon *Capitosauroides* cannot be definitively attributed to a temnospondyl amphibian trackmaker, although some features are consistent with this identification (e.g., absence of claw marks, wide-gauged gait). The wide-gauge gait and absence of claws, however, are not exclusive to temnospondyls. They are primitive features that are expected to be present in non-amniote tetrapods. Moreover, demonstrating the absence of claw marks can be quite difficult, requiring good preservation of several footsteps to offer the best opportunity to preserve those structures. We were not able to confirm that the above-mentioned *Capitosauroides* specimens definitively lack claws. The presence of a pentadactyl manus is inconsistent with a temnospondyl trackmaker, which would be expected to possess only four functional digits.

#### Moenkopi capitosaurid tracks

Peabody [30] described two isolated manus-pes pairs from the Early Triassic Moenkopi Formation of North America (Fig. 11C). The prints included only the tips of the toes, which were reported to lack claws, and were attributed to a capitosaurid trackmaker in part based on the presence of skeletal remains (UCMP 36718) collected from the same formation [30]. Capitosaurids are crocodile-like, aquatic temnospondyls that include forms such as *Mastodonsaurus* [31], the presumed German *Capitosauroides* trackmaker. The Moenkopi capitosaurid skeleton (UCMP 36713), which is now called *Wellesaurus peabodyi* [32], [33], was reported to have five digits on the manus [30]. The manus-pes pairs that Peabody attributed to a capitosaurid trackmaker were interpreted to have a pentadactyl manus and pes, like *Wellesaurus*. Notably, several of the preserved manual and pedal digit traces are associated with very narrow, straight drag marks.

The Moenkopi tracks were originally assigned to a temnospondyl trackmaker mainly because the presence of skeletal remains of these animals in the same stratigraphic levels. As occurs with *Capitosauroides*, the wide-gauged gait and the lack of claw marks are consistent with a temnospondyl trackmaker but do not provide a conclusive identification. The uncertainty surrounding the number of digits in the manus (see [30], pl. 27A), prevents unequivocally assigning the Moenkopi tracks to temnospondyl amphibians.

#### Paucity of Mesozoic Temnospondyl Tracks

Most of the Mesozoic ichnofossils attributed to temnospondyls are isolated tracks, and only a single trackway has been described. In all cases, the tracks appear to lack traces of claws and are quite small in comparison to *Episcopopus* (Fig. 5). In no case, however, could a tetradactyl manus be unambiguously identified. Rather, in most cases a pentadactyl condition was inferred. Apart from the Moenkopi capitosaurid skeleton, the only other putative pentadactyl temnospondyl described is the Australian stereospondyl *Paracyclotosaurus* [34]. Although it is not impossible that some temnospondyls had a pentadactyl manus, there is no specimen from the Mesozoic that definitively demonstrates the presence of five manual digits (CAM pers. obs.). Even if some basal temnospondyls did possess a pentadactyl manus, we consider it unlikely that the entirety of the temnospondyl track record would be made up of specimens possessing this condition.

In contrast, the Paleozoic temnospondyl footprint record includes a diverse collection of predominantly small tracks described from several Laurasian localities and stratigraphic levels extending from the Mississipian to the latest Permian [28,35-45]. All of these trackways indicate a trackmaker with a tetradactyl manus and a pentadactyl pes, which was considered diagnostic of “Amphibia” *sensu lato* or Temnospondyli [44]. In some cases, a temnospondyl trackmaker was inferred due to the relatively large size of the tracks [40]. Other referrals to temnospondyl trackmakers were based on correlation with the body fossil record [37], [39], [42], [43].

As previously mentioned, the paucity of footprints and trackways attributed to Mesozoic stereospondyls is striking in light of their relatively rich body fossil record, and several explanations are possible. Stereospondyls were predominantly semi-aquatic to aquatic tetrapods, with relatively weak limbs and probably limited capability of locomotion on land [21], [23], [46]. The low preservation potential of footprints in saturated sediments, as well as the poor registration of anatomical detail on such surfaces [47], probably contributes to their rarity. At Moyeni, the cross-cutting relationships with sedimentary structures, emergence features, and other biogenic traces indicate that the *Episcopopus* tracks were made after the ripple forms had stopped migrating, just after emergence of the bar surface. The unique track preservation and the plasticity of the sediment was probably the result of more than one factor (e.g., sediment composition and granulometry, absence of erosional processes) but certainly enhanced by the microbial mat that covered the bar top where the tracks are strikingly well preserved [4].

## Discussion

### Temnospondyl Locomotion

Salamanders have been regarded as the living animals that most closely resemble the ancestral condition for Tetrapoda in terms of locomotion due to their retention of sprawling gait with limbs of equal size, elongated trunk, and a tail [15,48-53]. Salamanders are usually capable of both aquatic and terrestrial locomotion. When walking on land, they have a sprawling posture and produce “standing waves” of body undulations that increase their stride length. When swimming, they use axial undulation of the trunk and tail to produce “travelling waves.” The limbs are held against the trunk to reduce drag and play no active role in propulsion [49], [54], [55].

According to the present understanding of the interrelationships of temnospondlys and their closest relatives [20], [56], stereospondyl temnospondyls are close relatives of crown-group amphibians (i.e., frogs, salamanders, caecilians). In this context, the Moyeni tracksite provides a rare opportunity to compare the locomotion of extinct tetrapods against a closely-related extant analogue. Nevertheless, it must be kept in mind that the *Episcopopus* trackway represents a single exemplar, made by an individual at one time in one place. The resultant ichnomorphology is a function of the interaction between the particular conditions of the Moyeni substrate (e.g., substrate wetness, inclination, grain size) and the *Episcopopus* trackmaker (e.g., body mass, morphology, behavior), as well as the subsequent modifications (e.g., taphonomic processes during burial and subsequent exposure). Taking into account all these considerations, the *Episcopopus* trackway preserved at Moyeni still differs in important ways from trackways made by living salamanders.

As previously mentioned, the *Episcopopus* trackway reveals a sprawling quadruped moving in a straight line. The footfall pattern and other trackway parameters are consistent across the 22 tracks scanned and studied here (Table S1). The underside of the body was not completely elevated from the substrate during locomotion, as evidenced by the well-marked body drag, which occupies a large part of the space between right and left manus and pes prints. None of the manus or pes prints overprint or were overprinted by the body drag, indicating that the limbs were directed laterally and the manus did not reach very far anteriorly during progression. There is little to no transverse movement captured by the body drag, tail drag, or pes drag. The lack of sinuosoidal movement in the pedal digit drags, which are straight and do not converge, and the regular, wide-gauge posture suggests that the rear part of the body did not undulate during locomotion. Moreover, the longitudinal grooves interpreted as part of the tail drag are also straight. In contrast, the arcuate manual digit drags indicate that at least part of the body, the pectoral girdle, was translated transversely during locomotion. The manus drags are asymmetrically S-shaped, with a short medial excursion preceding a longer, more arcuate lateral excursion. Successive manus drags form a more or less sinusoidal pattern whose amplitude indicates the extent of transverse movement of the shoulder girdle. Any sinusoidal movement of the pectoral region of the body might have been overprinted (and obscured) by the straight drag of the rear part of the body and tail.

Very similar drag marks were recorded [14] in trackways of the salamanders *Taricha* and *Triturus* (see [14], pl. 11), with the crucial difference that it is their pedes that make the sinusoidal drag marks and have a more regular footfall pattern than does the manus. In these and other living terrestrial sprawling tetrapods, both the forelimbs and hindlimbs have a similar weight-bearing capacity, but the hindlimbs are the primary propulsors. The forelimbs, in contrast, mainly provide deceleration during the step cycle [57-60]. Hind limb-driven locomotion depends on the size of the tail, which can produce important frictional forces [57], [58] that influence in the position of the center of mass in reptiles [59], [61]. In contrast to hind limb-driven locomotors, in semi-aquatic stereospondyls the center of mass was most probably positioned anteriorly due to the relatively larger skull, heavier pectoral girdle, and less massive pelvic girdle [22], [23]. Moreover, tail size relative to the body length (from the tip of the snout to the first caudal vertebrae) is substantially smaller in stereospondyls (ca. 50%, CAM pers. obs.) than the higher proportions (>80%) reported for salamanders and crocodiles [60] (Fig. 10). Together, the more forward position of the center of mass and the sinusoidal manus drags indicate that the forelimb, not the hindlimb, was the main propulsive element in the *Episcopopus* trackmaker, and possibly in other stereospondyls.

A second distinctive difference between *Episcopopus* and modern salamander tracks is that the former lacks impressions of the proximal phalanges and metapodials. The *Episcopopus* manus and pes prints are well-marked but consist of only the tips of digits, all of which lack claw marks as well as any evidence of cornified structures that are present occasionally in some salamander and anuran genera [62]. The absence of proximal phalanges and metapodials in the manus and pes prints may reflect a difference related to the substrate condition, behavior, or morphology. Temnospondyl skeletons (Paleozoic and Mesozoic) rarely preserve ossified carpals and tarsals, although they are present in certain heavily ossified forms that have been interpreted to be more terrestrial [23], [63]. Nevertheless, most Paleozoic footprints and trackways attributed to temnospondyls show conspicuous palm and sole impressions, and variations appear to be related to substrate consistency [35,37-41,44,45]. In living salamanders (e.g., *Triturus torosus*) imprinting of the manus and pes also varies with substrate consistency, as does the presence of drag marks, particularly on saturated sediments (e.g., UCMP V4133-36752 and V4133-36745; [14]). On soft or slippery surfaces, *Triturus torosus* leaves drags marks when the manus/pes sink more deeply in the sediment during the “stance phase” of the step cycle [14], [64]. In contrast, “tips only” prints are associated with firm or cohesive substrates, where only the part of the autopodium that exerts the most pressure on the substrate, in this case the tips of the digits, is imprinted [14], [64].

According to our investigations of the Moyeni tracksite, at the time of imprinting, the surface of the emergent part of the sand bar was moist but not saturated and covered in a microbial mat [4]. Therefore, we consider that this unique trackway morphology reflects the ability of the *Episcopopus* trackmaker to “pull” its body over the slippery microbial-matted surface using its forelimbs as the main propulsive elements, with the hindlimbs mainly giving support to the rear part of body and tail. We propose that this form of forelimb-driven locomotion allowed these large, heavy, semi-aquatic animals with relatively weak limbs to engage in intermittent bouts of terrestrial locomotion.

### Implications for basal tetrapod locomotion

Recent discussions of ancient tetrapod locomotion focused on the sequence of changes in the fins and limbs during the transition from swimming to walking on land [65], [66], specifically on which of the two sets of paired appendages was first enlisted in terrestrial locomotion. King and colleagues [65] argued that the use of pelvic appendages as major propulsors as a characteristic of terrestrial tetrapod locomotion arose in sarcopterigian fishes, before the evolution of digited limbs. In contrast, Pierce and colleagues [66] in their studies on *Ichthyostega* locomotor performance suggested that the forelimb was the first enlisted in land locomotion, with hindlimb-powered terrestrial locomotion acquired in later stages of tetrapod evolution. These authors [66] suggested that because the hindlimbs of Devonian stem-tetrapods were adapted for swimming, rather than for interaction with the substrate, they were unable to employ a modern tetrapod quadrupedal progression on land. They [66], [67] concluded that salamanders might be appropriate “model organisms” for the study of locomotor function in crown-group tetrapods, but they might not be reliable for early stages of the water-land transition in stem-tetrapods.

Fossil tracks and trackways, although preserving only a small portion of trackmaker anatomy, can under certain conditions provide more direct evidence of paleobiology than can be inferred from the fossil body record. This strength is balanced by the taxonomic coarseness with which trackmakers can be identified using synapomophies of body fossil taxa [5], [17]. In the present case, the *Episcopopus* trackmaker has been interpreted to be a relatively large, semi-aquatic stereospondyl that was moving along the top of a partially emerged sand bar at the bend in a river. The animal progressed with a sprawling posture, using a lateral-sequence walk without a substantial bending of the trunk. Our analysis suggests that its forelimbs were the major propulsive elements, which dramatically differs from hindlimb-driven locomotion of salamanders and other extant terrestrial tetrapods. If this is true, then salamanders might not be good “model organisms” to study the locomotor behavior of early aquatic and semi-aquatic crown-group tetrapods. Stereospondyls were suitable to perform quadrupedal walking on land, as other sprawling extant tetrapods (i.e. salamanders), but differences in their skeletal plan that likely influence the position of the center of mass resulted in completely different locomotor behaviors (Fig. 10). Further detailed investigations on Paleozoic trackways, in concert with studies of locomotor performance in fossil basal tetrapods, will shed light on the question of how and when the locomotor behavior observed in extant terrestrial sprawling tetrapods evolved.

## Conclusions

Approximately 200 million years ago in the part of western Gondwana that is now the southern African country of Lesotho, a partially emerged, microbial-matted sand bar on the inner bank of a river bend was repeatedly traversed by dinosaurs and other tetrapods. The first animal to make tracks across the bar soon after it was subaerially exposed a large stereospondyl amphibian. The tracks, known as *Episcopopus ventrosus*, represent one of the only definitive temnospondyl trackways known from the Mesozoic Era. The Mesozoic record of temnospondyl tracks is extremely sparse compared to their Paleozoic record, which is relatively much more abundant and spatiotemporally wide-ranging. One possible explanation for this disparity between Paleozoic and Mesozoic track abundance is that the temnospondyls from these respective eras differed in their terrestrial capabilities. Large temnospondyls of the Mesozoic have been inferred to be more aquatic, based on their flattened skulls with dorsally-facing eyes and their poorly ossified distal limb skeleton.

The *Episcopopus* trackway records a 3.5 m long animal dragging its belly across a wet substrate. In addition to a well-marked body drag, manual digits recorded sinuous drag marks. A detailed study of the trackway has led us to conclude that the animal was reaching with the tips of its fingers to pull its body over the surface and probably using the slippery wet surface to facilitate its progression. This locomotor behavior differs from those observed in living salamanders moving on slippery surfaces, which has been used as living models to interpret basal tetrapod locomotion. The differences between temnospondyl locomotion inferred from trackways and observed salamander locomotion can be explained by major differences in the body shape, size, and skeletal plan of these two groups, which strongly influenced their terrestrial capabilities.

## Supporting Information

Table S1 **Measurements of** ***Episcopopus*** **trackway at Moyeni, Lesotho.** Numbers identifying the successive manus (**m**) and pes (**p**) in the trackway follow original Ellenberger' [3] numbering. Linear measurements are expressed in centimeters and angles in degrees; em-dash denotes missing *datum* due to preservation bias.
(PDF)

## References

[1] Olsen PE, Galton PM (1984) A review of the reptile and amphibian assemblages from the Stormberg of southern Africa, with special emphasis on the footprints and the age of the Stormberg. Palaeontologia Africana 25: 87–110.

[2] Olsen PE, Sues H-D (1986) Correlations of the continental Late Triassic and Early Jurassic sediments, and patterns of the Triassic-Jurassic tetrapod transition. In: Padian K, editor. The Beginning of the Age of Dinosaurs. Cambridge: Cambridge University Press. pp. 321–351.

[3] Ellenberger P (1974) Contribution à la classification des pistes de vertèbres du Trias: les types du Stormberg d'Áfrique du Sud (II partie): le Stormberg Superieur-I. Le biome de la zone B/1 ou niveau de Moyeni: ses biocenoses). Palaeovertebrata Mémoire Extraordinaire: 1–202

[4] Smith RMH, Marsicano CA, Wilson JA (2009) Sedimentology and paleoecology of a diverse Early Jurassic tetrapod tracksite in Lesotho, southern Africa. Palaios 24: 672–684.

[5] Wilson JA, Marsicano CA, Smith RMS (2009) Dynamic locomotor capabilities revealed by early dinosaur trackmakers from southern Africa. PLoS One 4: e7331. 19806213 10.1371/journal.pone.0007331 PMC2752196

[6] Ellenberger P (1970) Les niveaux paleontologiques de premiere apparition des mammiferes primordiaux en Afrique du Sud et leur ichnologie. Establissement de zones stratigraphiques detaillees dans le Stormberg de Lesotho (Afrique du Sud) (Trias Superieur a Jurassique). Proceedings II Gondwana Symposium, Pretoria 2: 340–370.

[7] Lucas SG (2008) Global Jurassic tetrapod biochronology. Volumina Jurassica 6: 99–108.

[8] Porchetti SDO, Nicosia U (2007) Re-Examination of Some Large Early Mesozoic Tetrapod Footprints from the African Collection of Paul Ellenberger. Ichnos 14: 219–245.

[9] Knoll F (2005) The tetrapod fauna of the Upper Elliot and Clarens formations in the main Karoo Basin (South Africa and Lesotho). Bulletin de la Société Géologique de France 176: 81–91.

[10] Requirand C (2001) Catalogue des pistes du Stormberg (Lesotho). Collection Paul Ellenberger: Université Montpellier II. Institut de Sciences de l'Evolution, Laboratoire de Paléontologie.

[11] Prave AR (2002) Life on land in the Proterozoic: evidence from the Torridonian rocks of northwest Scotland. Geology 30: 811–814.

[12] Porada H, Gherbut J, Bouougri EH (2008) Kinneyia-type wrinkle structures—critical review and model of formation. Palaios 23: 65–77.

[13] Noffke N (2009) The criteria for the biogeneicity of microbially induced sedimentary structures (MISS) in Archean and younger, sandy deposits. Earth-Science Reviews 96: 173–180.

[14] Peabody FE (1959) Trackways of living and fossil salamanders. University of California Publications in Zoology 63: 1–72.

[15] Ashley-Ross MA, Bechtel BF (2004) Kinematics of the transition between aquatic and terrestrial locomotion in the newt *Taricha torosa* . Journal of Experimental Biology 207: 461–474. 14691094 10.1242/jeb.00769

[16] Kitching JW, Raath MA (1984) Fossils from the Elliot and Clarens Formation (Karoo sequence) of the northeastern cape, Orange Free State and Lesotho, and a suggested biozonation based on tetrapods. Palaeontologia Africana 24: 111–125.

[17] Carrano MT, Wilson JA (2001) Taxon distributions and the tetrpod track record. Paleobiology 27: 564–582.

[18] Hutchinson JR, Gatesy SM (2000) Adductors, abductors, and the evolution of archosaur locomotion. Paleobiology 26: 734–751.

[19] Ruta M, Coates MI, Quicke DLJ (2003) Early tetrapod relationships revisited. Biological Reviews 78: 251–345. 12803423 10.1017/s1464793102006103

[20] Ruta M, Coates MI (2007) Dates, nodes and character conflict: addressing the lissamphibian origin problem. Journal of Systematic Palaeontology 5: 69–122.

[21] Warren AA (2000) Secondary aquatic temnospondyls from the Upper Permian and Mesozoic. In: Heatwole H, Carroll R, editors. Amphibian Biology: Volume 4 Paleontology: The Evolutionary History of Amphibians. pp. 1121–1149.

[22] Warren AA, Hutchinson MN (1983) The last labyrinthodont? A new brachypoid (Amphibia, Temnospondyli) from the Early Jurassic Evergreen Formation of Queensland. Philosophical Transactions of the Royal Society B 303: 1–62.

[23] Schoch RR, Milner AR (2000) Stereospondyli. Munich: Verlag Dr. Friedrich Pfeil. 203 p.

[24] Warren AA, Damiani RJ (1999) Stereospondyl amphibians from the Elliot Formation of South Africa. Paleontologia Africana 35: 45–54.

[25] Pohlig H (1893) Vorlage von Flieβpapierabdrücken dossiler Wirbeltierfuβstapfen aus dem Buntsandstein von Hildburghausen und Bernburg. Verhandlungen des Naturhistorischen Vereines der preussischen Rheinlande und Westphalens 50: 82–83.

[26] Spitz W (1923) Notiz über eine Labyrinthodontenfährte aus der Trias. Paläontologische Zeitschrift 5: 380–381.

[27] Haubold H (1971) Die Tetrapodenfa-hrten des Buntsandsteins. Paläontologische Abhandlungen A 4: 395–548.

[28] Haubold H (1971) Ichnia Amphibiorum et Reptiliorum fossilium. Handbuch der Pala-oherpetologie 18: 1–124.

[29] Fuglewicz R, Ptaszyiqski T, Rdzanek K (1990) Lower Triassic footprints from the Swiqtokrzyskie (Holy Cross) Mountains, Poland. Acta Palaeontologica Polonica 35: 109–164.

[30] Peabody FE (1948) Reptile and amphibian trackways from the Lower Triassic Moenkopi Formation of Arizona and Utah. Bulletin of the Department of Geological Sciences, University of California Publications 27: 295–467.

[31] Schoch RR (1999) Comparative osteology of *Mastodonsaurus giganteus* (Jaeger, 1828) from the Middle Triassic (Lettenkeuper: Longobardian) of Germany (Baden-Württemberg, Bayern, Thüringen). Stuttgarter Beiträge zur Naturkunde Serie B (Geologie und Paläontologie) 278: 1–175.

[32] Welles SP, Cosgriff JW (1965) A revision of the labyrinthodont family Capitosauridae and a description of *Parotosaurus peabodyi* n. sp. from the Moenkopi Formation of Northern Arizona. University of California Publications in Geological Sciences 54: 1–148.

[33] Lehman JP (1971) Nouveaux vertébrés fossiles du trias de la serie de Zarzaïtine. Annales de Paléontologie (Vertébrés) 57: 71–93.

[34] Watson DMS (1958) A new labyrinthodont (*Paracyclotosaurus*) from the Upper Trias of New South Wales. Bulletin of the British Museum of Natural History, Geology 3: 233–263.

[35] Baird D (1952) Revision of the Pennsylvanian and Permian footprints *Limnopus*, *Allopus* and *Baropus* . Journal of Paleontology 26: 832–840.

[36] Braddy SJ, Morrisey LB, Yates AM (2003) Amphibian swimming traces from the lower Permian of New Mexico. Palaeontology 46: 671–683.

[37] Haubold H, Allen A, Prescott Atkinson T, Buta RJ, Lacefield JA (2005) Interpretation of the tetrapod footprints from the Early Pennsylvanian of Alabama. In: Buta RJ, Rindberg, A.K, Kopaska-Merkel, D.C., editor. Pennsylvanian Footprints in the Black Warrior Basin of Alabama. pp. 75–111.

[38] Minter NJ, Braddy SJ, Davis RB (2007) Between a rock and a hard place: arthropod trackways and ichnotaxonomy. Lethaia 40: 365–375.

[39] Falcon-Lang HJ, Gibling MR, Benton MJ, Miller RF, Bashforth AR (2010) Diverse tetrapod trackways in the Lower Pennsylvanian Tynemouth Creek Formation, near St. Martins, southern New Brunswick, Canada. Palaeogeography, Palaeoclimatology, Palaeoecology 296: 1–13.

[40] Lucas SG, Fillmore D, Simpson E (2010) The Mississippian tetrapod footprint ichnogenus *Palaeosauropus*: extramorphological variation and ichnotaxonomy. Ichnos 17: 177–186.

[41] Ptaszyński T, Niedźwiedzki G (2004) Late Permian vertebrate tracks from the Tumlin Sandstone, Holy Cross Mountains Poland. Acta Paleontologica Polonica 49: 289–320.

[42] Sarjeant W, Mossman D (1974) Vertebrate footprints from the Carboniferous sediments of Nova Scotia: a historical review and description of newly discovered forms. Palaeogeography, Palaeoclimatology, Palaeoecology 23: 279–306.

[43] Turek V (1989) Fish and amphibian trace fossils from the Westphalian sediments of Bohemia. Palaeontology 32: 623–643.

[44] Scarboro D, Tucker M (1995) Amphibian footprints from the mid-Carboniferous of Northumberland, England: sedimentological context, preservation and significance. Palaeogeography, Palaeoclimatology, Palaeoecology 113: 335–349.

[45] Voight S, Saber H, Schneider J, Hmich D, Hminna A (2011) Late Carboniferous-Early Permian Tetrapod Ichnofauna from the Khenifra Basin, Central Morocco. Geobios 44: 399–407.

[46] Carroll R (2009) The Rise of Amphibians: 365 Million Years of Evolution. Baltimore, Maryland USA: John Hopkins University Press.

[47] Milàn J (2006) Variations in the morphology of emu (*Dromaius novaehollandiae*) tracks, reflecting differences in walking pattern and substrate consistency: ichnotaxonomical implications. Palaeontology 49: 405–420.

[48] Schaeffer B (1941) The morphological and functional evolution of the tarsus in amphibians and reptiles. Bulletin of the American Museum of Natural History LXXVIII: 395–472.

[49] Edwards JL (1989) Two perspectives on the evolution of the tetrapod limb. American Zoologist 29: 235–254.

[50] Gray J (1944) Studies in the mechanics of the tetrapod skeleton. Journal Experimental Biology 20: 88–116. 10.1242/jeb.000687 17601942

[51] Reilly SM, McElroy EJ, Odum RA, Hornyak VA (2006) Tuataras and salamanders show that walkingand running mechanics are ancientfeatures of tetrapod locomotion. Proceedings of the Royal Society of London B. 10.1098/rspb.2006.3489 PMC1560307 16777753

[52] Ashley-Ross M, Lundin R, Johnson J (2009) Kinematics of level terrestrial and underwater walking in the california newt, *Taricha torosa* . Journal of Experimental Zoology 311A: 240–257. 10.1002/jez.522 19266497

[53] Horner AM, Jayne BC (2014) Lungfish axial muscle function and the vertebrate water to land transition. PLoS ONE 9(5): e96516 doi:10.1371/journal.pone.0096516 24788982 10.1371/journal.pone.0096516 PMC4008594

[54] Delvolvé I, Bem T, Cabelguen J-M (1997) Epaxial and limb muscle activity during swimming and terrestrial stepping in the adult newt, *Pleurodeles waltl* . Journal of Neurophysiology 78: 638–650. 9307101 10.1152/jn.1997.78.2.638

[55] Frolich LM, Biewener AA (1992) Kinematic and electromyographic analysis of the functional role of the body axis during terrestrial and aquatic locomotion in the salamander *Ambystoma tigrinum* . Journal of Experimental Biology 162: 107–130.

[56] Maddin HC, Jenkins FA Jr, Anderson JS (2012) The braincase of *Eocaecilia micropodia* (Lissamphibia, Gymnophiona) and the origin of caecilians. PLoS ONE 7(12): e50743 doi:10.1371/journal.pone.0050743 23227204 10.1371/journal.pone.0050743 PMC3515621

[57] Ashley-Ross MA (1994) Hindlimb kinematics during terrestrial locomotion in a salamander (*Dicamptodon tenebrosus*). Journal of Experimental Biology 193: 255–283. 9317755 10.1242/jeb.193.1.255

[58] Blob RW, Biewener AA (2001) Mechancs of limb bone loading during terrestrial locomotion in the green iguana (*Iguana iguana*) and American alligator (*Alligator mississippiensis*). Journal of Experimental Biology 204: 1099–1122. 11222128 10.1242/jeb.204.6.1099

[59] Willey JS, Biknevicius AR, Reilly SM, Earls KD (2004) The tale of the tail: limb function and locomotor mechanics in *Alligator mississippiensis* . Journal of Experimental Biology 207: 553–563. 14691103 10.1242/jeb.00774

[60] Kawano SM, Blob RW (2013) Propulsive forces of mudskipper fins and salamander limbs during terrestrial locomotion: implications for the invasion of land. Integrative and Comparative Biology 53: 283–294. 23667046 10.1093/icb/ict051

[61] Allen V, Paxton H, Hutchinson JR (2009) Variation in Center of Mass Estimates for Extant Sauropsids and its Importance for Reconstructing Inertial Properties of Extinct Archosaurs. The Anatomical Record 291: 1442–1461. 10.1002/ar.20973 19711477

[62] Maddin HC, Eckhart L, Jaeger K, Russell AP, Ghannadan M (2009) The anatomy and development of the claws of *Xenopus laevis* (Lissamphibia: Anura) reveal alternate pathways of structural evolution in the integument of tetrapods. Journal of Anatomy 214: 607–619. 19422431 10.1111/j.1469-7580.2009.01052.x PMC2736125

[63] Pawley K, Warren A (2006) The appendicular skeleton of *Eryops megacephalus* Cope, 1877 (Temnospondyli: Eryopoidea) from the Lower Permian of North America. Journal of Paleontology 80: 561–580.

[64] Brand L (1996) Variations in salamander trackways resulting from substrate differences. Journal of Paleontology 70: 1004–1010.

[65] King HM, Shubin NH, Coates MI, Hale ME (2011) Behavioral evidence for the evolution of walking and bounding before terrestriality in sarcopterygian fishes. Proceedings of the National Academy of Sciences 108: 21146–21151. 10.1073/pnas.1118669109 PMC3248479 22160688

[66] Pierce SE, Clack JA, Hutchinson JR (2012) Three-dimensional limb joint mobility in the early tetrapod Ichthyostega. Nature 486: 523–526. 22722854 10.1038/nature11124

[67] Pierce SE, Hutchinson JR, Clack JA (2013) Historical perspectives on the evolution of tetrapodomorph movement. Integrative and Comparative Biology 53: 209–223. 23624864 10.1093/icb/ict022

