# Supplementary material for: A Temnospondyl Trackway from the Early Mesozoic of Western Gondwana and Its Implications for Basal Tetrapod Locomotion
Source: PLoS One. 2014 Aug 6;9(8):e103255. doi: 10.1371/journal.pone.0103255 (PMC4123899; doi:10.1371/journal.pone.0103255)
Supplement: Table S1 — Measurements of Episcopopus trackway at Moyeni, Lesotho. Numbers identifying the successive manus (m) and pes (p) in the trackway follow original Ellenberger' [3] numbering. Linear measurements are expressed in centimeters and angles in degrees; em-dash denotes missing datum due to preservation bias. (PDF) [file pone.0103255.s001.pdf]

Table S1. Measurements of *Episcopopus* trackway at Moyeni, Lesotho.
Stride length is measured as the straight-line distance between homologous points on successive footfalls of the same foot. Pace length is the straight line distance between homologous points on left and right manus (m) or pes (p) prints. Successive pace lengths (i.e., Right–Left–Right or Left–Right–Left) form an angle that is measured as pace angulation for both pes and manus. Linear measurements were made to the nearest 0.5 centimeter and were measured *in situ* unless noted; angular measurements were made to the nearest 0.5 degree using the digital map of the trackway surface.

Left	Right	prints		trackway		
		length	width	pace length (cm)	stride length (cm)	angle (degrees)
	**70p**	—	—	95	100	65
	**70m**	10	22	90	95	60
**69p**		—	—	95	110	85
**69m**		10	20	95	105	60
	**68p**	12	15	90	—	—
	**68m**	—	—	87	117	60
**67p**		18	20	—	110	—
**67m**		9	24	105	110	65
	**66p**	—	—	—	—	—
	**66m**	12	20	95	—	—
**65p**		17	22	120	117	—
**65m**		15	20	110	—	—
	**64p**	—	—	—	—	—
	**64m**	—	—	—	—	—
**63p**		16	23	—	105	—
**63m**		—	—	—	—	—
	**62p**	—	—	—	—	—
	**62m**	—	—	96	—	—
**61p**		15	20	—	105	—
**61m**		13	22	—	105	—
	**60p**	—	—	—	—	—
	**60m**	—	—	—	—	—
**59p**		16	22	95	113	80
**59m**		12	20	98	116	70
	**58p**	—	—	92	117	75
	**58m**	15	20	85	115	68
**57p**		20	23	102	115	65
**57m**		—	—	105	107	65
	**56p**	15	25	95	—	—
	**56m**	—	—	89	115	70
**55p**		—	—	—	—	—
**55m**		14	20	100	105	70
	**54p**	—	—	—	—	—
	**54m**	15	19	85	105	70
**53p**		—	—	—	—	—
**53m**		—	—	95	87	65
	**52p**	—	—	—	—	—

	**52m**	—	—	82	106	70
**51p**		—	—	—	—	—
**51m**		20	20	120	130	80
	**50p**	17	18	115	-	-
	**50m**	—	—	95	-	-
**49p**		17	18	-	-	-
**49m**		—	—	-	-	-
**Average**		m = 12.3	m = 20.5	97,3	109,1	69,1
		p = 16.5	p = 22.8			

Numbers identifying the successive manus (m) and pes (p) in the trackway follow original Ellenberger's [3] numbering. Linear measurements are expressed in centimeters and angles in degrees; em-dash denotes missing datum due to preservation bias.
